# Supplementary material for: Perspectives on physics-based one-dimensional modeling of lung physiology
Source: Front Physiol. 2025 Sep 24;16:1635983. doi: 10.3389/fphys.2025.1635983 (PMC12504317; doi:10.3389/fphys.2025.1635983)
Supplement: Supplementary file 1 [file DataSheet1.pdf]

## Supplementary Materials
## for
# Perspectives on physics-based one-dimensional modeling of lung physiology

Aranyak Chakravarty,[1,2] Debjit Kundu,[2] Mahesh V. Panchagnula,[2] Alladi Mohan,[3] and Neelesh A. Patankar[4,a)]

[1)] *School of Nuclear Studies & Application, Jadavpur University, Kolkata, India*

[2)] *Department of Applied Mechanics & Biomedical Engineering, Indian Institute of Technology Madras, Chennai, India*

[3)] *Department of Medicine, Sri Venkateswara Institute of Medical Sciences, Tirupati, India*

[4)] *Department of Mechanical Engineering, Northwestern University, Evanston, IL, USA*

[a)] Electronic mail: n-patankar@northwestern.edu

## S1. 1D ANALYTICAL SOLUTION: EXPONENTIAL VARIATION

This section details the analytical approach for obtaining solution of Eq. 15 considering an exponential variation of the lung cross-sectional area. The total cross-sectional area ($A_x$) along the length ($x$) of the idealised lung geometry is approximated using an exponential function as

$$A_x = A_0 \text{exp}(\beta x), \tag{S1}$$

where $A_0$ is the cross-sectional area at $x = 0$ and $\beta$ is the area-change factor. Integrating Eq. 15, we obtain -

$$\Big(Qc_g - A_x D_g \frac{\partial c_g}{\partial x}\Big) = K_1, \tag{S2}$$

where $K_1$ is the integration constant. Dividing the above equation with $A_x D_g$ and rearranging using Eq.S1, we obtain -

$$\frac{\partial c_g}{\partial x} - \frac{Q}{A_0 \text{exp}(\beta x) D_g} c_g = -\frac{K_1}{A_0 \text{exp}(\beta x) D_g}, \tag{S3}$$

which is of the form $y' + py = q$. The integrating factor for Eq. S3 is evaluated as

$$l = \text{exp}\Bigg[\int p dx\Bigg] = \text{exp}\Bigg[-\frac{Q}{\beta A_0 D_g}\text{exp}(-\beta x)\Bigg] = \text{exp}\Bigg[b\text{exp}(-\beta x)\Bigg], \tag{S4}$$

where $b = -\dfrac{Q}{\beta A_0 D_g}$. Utilising this, the integration is carried out as follows -

$$c_{g,x} l = \int l q dx, \tag{S5}$$

$$\begin{aligned} \implies c_{g,x}\text{exp}\Big[b\text{exp}(-\beta x)\Big] &= \int \text{exp}\Big[b\text{exp}(-\beta x)\Big]\Bigg(-\frac{K_1}{A_0\text{exp}(\beta x) D_g}\Bigg) dx \\ &= -\frac{K_1}{A_0 D_g}\int \text{exp}\Big[b\text{exp}(-\beta x) - \beta x\Big] dx, \end{aligned} \tag{S6}$$

$$\implies c_{g,x}\text{exp}\Big[b\text{exp}(-\beta x)\Big] = \frac{K_1}{A_0 D_g}\frac{1}{\beta b}\text{exp}\Big[b\text{exp}(-\beta x)\Big] + K_2, \tag{S7}$$

$$\begin{aligned} \implies c_{g,x} &= \Bigg[\frac{K_1}{Q}\text{exp}\Big[b\text{exp}(-\beta x)\Big] + K_2\Bigg]\text{exp}\Big[-b\text{exp}(-\beta x)\Big], \\ &= \frac{K_1}{Q} + K_2\text{exp}\Big[-b\text{exp}(-\beta x)\Big]. \end{aligned} \tag{S8}$$

Equation S8 is solved subject to the following boundary conditions (see assumptions) -

$$c_{g,x}|_{x=0} = c_{g,0}, \tag{S9}$$

$$K_1|_{x=L} = A_L D_{ex}(c_{g,L} - c_{g,\infty}). \tag{S10}$$

Utilising the boundary conditions, we can write -

$$c_{g,0} = \frac{K_1}{Q} + K_2 \text{exp}(-b) = \frac{K_1}{Q} + K_2 E_0, \tag{S11}$$

$$K_1 = \frac{A_L D_{ex}\left[c_{g,0}\frac{E_L}{E_o} - c_{g,\infty}\right]}{\left[1 - \frac{A_L D_{ex}}{Q}\left(1 - \frac{E_L}{E_0}\right)\right]}, \tag{S12}$$

where $E_0$ and $E_L$ represents the exponential terms in the above equations at $x = 0$ and $x = L$, respectively. Equation S12 is re-arranged using Eq. S1 to obtain the final expression of $K_1$ as

$$K_1 = \frac{A_L D_{ex}\left[c_{g,0} - c_{g,\infty}\frac{E_0}{E_L}\right]}{\left[\frac{E_0}{E_L} - \frac{A_L D_{ex}}{Q}\frac{E_0}{E_L} + \frac{A_L D_{ex}}{Q}\right]} = \frac{Q\left[c_{g,0} - c_{g,\infty}\frac{E_0}{E_L}\right]}{\left[\frac{Q}{A_0 D_{ex}}\frac{E_0}{E_L \text{exp}(-\beta L)} - \frac{E_0}{E_L} + 1\right]}. \tag{S13}$$

$K_2$ is determined using Eq. S11 as

$$K_2 = c_{g,0} - \frac{K_1}{Q}E_0. \tag{S14}$$

Substituting the expressions of $K_1$ and $K_2$ in Eq. S8 gives us the final solution in terms of $c_{g,x}$. This is expressed considering $c_{g,\infty} \to 0$ as follows

$$\begin{aligned}\lim_{c_{g,\infty},0}\frac{c_{g,x}}{c_{g,0}} = \phi_{g,x} &= 1 + \frac{\left(\frac{E_0}{E_L} - \frac{E_x}{E_L}\right)\left[1 - \frac{Z}{\text{exp}(-\beta L)}\right]}{1 - \frac{E_0}{E_L} + \frac{Z E_0}{E_L \text{exp}(-\beta L)}} \\ &= 1 + \frac{\left[\text{exp}\left[Pe_{g,e}\left(1 - e^{-\beta L}\right)\right] - \text{exp}\left[Pe_{g,e}\left(e^{-\beta x} - e^{-\beta L}\right)\right]\right]\left[1 - \frac{Z}{e^{-\beta L}}\right]}{1 - \text{exp}\left[Pe_{g,e}\left(1 - e^{-\beta L}\right)\right]\left[1 - \frac{Z}{e^{-\beta L}}\right]},\end{aligned} \tag{S15}$$

where $Pe_{g,e}$ $\left(= \dfrac{QL_0}{A_0 D_g}\right)$ and $Z$ $\left(= \dfrac{Q}{A_0 D_{ex}}\right)$ represents the Peclet number for gases (exponential variation) and the gas exchange parameter with the blood stream, respectively.

## S2. 1D ANALYTICAL SOLUTION: POWER LAW VARIATION

The length ($L_N$) and the total cross-sectional area ($A_N$) at each generation ($N$) of the idealised lung geometry can be approximated using a power-law function (see Section 1: Fig. 2 and Eq. 1). The length-change ($\alpha$) and area-change ($\beta$) factors in Eq. 1 are selected such that the computed length and area at each generation matches Weibel's morphometric data[1] as closely as possible (see Fig. 2a). The airway length ($x$), in terms of the lung generation number $N$, is given by

$$x_N = \frac{L_0(1-\alpha^{N+1})}{1-\alpha}. \tag{S16}$$

Since the length and area variation is assumed to be a function of $N$, the steady-state gas transport equation (Eq. 15) is re-written in terms $N$ as

$$\frac{\partial}{\partial N}\left(Qc_{g,N} - A_N D_g H_N \frac{\partial c_{g,N}}{\partial N}\right) = 0, \tag{S17}$$

using $H_N = \dfrac{\partial N}{\partial x_N}$. It is to be noted that although $N$ is an integer, it is treated as a continuous variable in all transport equations for computational convenience. Integrating, we obtain

$$\left(Qc_{g,N} - A_N D_g H_N \frac{\partial c_{g,N}}{\partial N}\right) = K_1, \tag{S18}$$

where $K_1$ is the integration constant. Dividing the above equation with $A_N D_g H_N$ and rearranging using Eq. 1, we obtain

$$\frac{\partial c_{g,N}}{\partial N} - \frac{Q}{A_0(2\beta)^N D_g H_N} c_{g,N} = -\frac{K_1}{A_0(2\beta)^N D_g H_N}, \tag{S19}$$

which is of the form $y' + py = q$ similar to that obtained for exponential variation. The solution technique used is also similar to that used for exponential variation. The integrating factor is evaluated as

$$l = \exp\left[\int p dN\right] = \exp\left[\int -\frac{Q}{A_0(2\beta)^N D_g H_N} dN\right] = \exp\left[-\frac{Q}{A_0(2\beta)^N D_g H'} \frac{(\alpha/2\beta)^N}{\ln(\alpha/2\beta)}\right] = \exp[ba^N], \tag{S20}$$

where $H' = \dfrac{1-\alpha}{-L_0\alpha\ln(\alpha)\alpha^N}$, $a = \alpha/2\beta$ and $b = -\dfrac{Q}{A_0(2\beta)^N D_g H'\ln(\alpha/2\beta)}$. Utilising this, the integration is carried out as follows -

$$c_{g,N}l = \int lq dN, \tag{S21}$$

$$\implies c_{g,N}\exp\Big[ba^N\Big] = \int \exp\Big[ba^N\Big]\left(-\frac{K_1}{A_0(2\beta)^N D_g H_N}\right)dN = -\frac{K_1}{A_0 D_g H'}\int a^N \exp\Big[ba^N\Big]dN, \tag{S22}$$

$$\implies c_{g,N}\exp\Big[ba^N\Big] = -\frac{K_1}{A_0 D_g H'}\frac{\exp\Big[ba^N\Big]}{b\ln(a)} + K_2, \tag{S23}$$

$$\begin{aligned}\implies c_{g,N} &= \left[-\frac{K_1}{A_0 D_g H'}\frac{\exp\Big[ba^N\Big]}{b\ln(a)} + K_2\right]\exp\Big[-ba^N\Big],\\ &= \left[-\frac{K_1}{A_0 D_g H'}\frac{A_0 D_g H'\ln(\alpha/2\beta)}{-Q\ln(\alpha/2\beta)}\right] + K_2\exp\Big[-ba^N\Big],\\ &= \frac{K_1}{Q} + K_2\exp\left[-\frac{QL_0\alpha\ln(\alpha)}{A_0 D_g(1-\alpha)\ln(\alpha/2\beta)}\left(\frac{\alpha}{2\beta}\right)^N\right].\end{aligned} \tag{S24}$$

Equation S24 is solved subject to the boundary conditions discussed before (see assumptions). Mathematically, these boundary conditions are expressed in terms of $N$ as -

$$c_{g,N}|_{N=0} = c_{g,0}, \tag{S25}$$

$$K_1|_{N=M} = A_M D_{ex}(c_{g,M} - c_{g,\infty}), \tag{S26}$$

where $M = 23$ i.e. the terminal lung generation. Utilising the boundary conditions, we can write

$$c_{g,0} = \frac{K_1}{Q} + K_2\exp\left[-\frac{QL_0\alpha\ln(\alpha)}{A_0 D_g(1-\alpha)\ln(\alpha/2\beta)}\left(\frac{\alpha}{2\beta}\right)^0\right] = \frac{K_1}{Q} + K_2 E_0, \tag{S27}$$

$$\implies K_1 = A_M D_{ex}\left[c_{g,0}\frac{E_M}{E_o} - c_{g,\infty}\right]\left[1 - \frac{A_M D_{ex}}{Q}\left(1 - \frac{E_M}{E_0}\right)\right], \tag{S28}$$

where $E_M$ and $E_0$ represents the exponential term in Eq. S27 for at $N = M$ and $N = 0$, respectively. Equation S28 is re-arranged using Eq. **??** to obtain the final expression of $K_1$ as

$$K_1 = A_M D_{ex}\left[c_{g,0}\frac{E_M}{E_o} - c_{\inf}\right]\left[1 - \frac{A_M D_{ex}}{Q}\left(1 - \frac{E_M}{E_0}\right)\right]. \tag{S29}$$

We define the following

$$\delta = \frac{\alpha}{2\beta}, Pe_{g,pl} = \frac{QL_0\alpha\ln(\alpha)}{A_0 D_g(1-\alpha)\ln(\alpha/2\beta)}, Z = \frac{Q}{A_0 D_{ex}}, \tag{S30}$$

such that we can write $E_N = \exp(-Pe_{g,pl}\delta^N)$. Eq. S29 can, thus, be written as

$$K_1 = A_M D_{ex}\left[c_{g,0}\frac{\exp(-Pe_{g,pl}\delta^M)}{\exp(-Pe_{g,pl}\delta^0)} - c_{g,\infty}\right]\left[1 - \frac{A_M D_{ex}}{Q}\left(1 - \frac{\exp(-Pe_{g,pl}\delta^M)}{\exp(-Pe_{g,pl}\delta^0)}\right)\right], \tag{S31}$$

which can further be re-arranged using Eq. **??** as

$$K_1 = Q\left[c_{g,0} - c_{g,\infty}\exp\Big(Pe_{g,pl}(\delta^M - 1)\Big)\right]\left[1 - \exp\Big(Pe_{g,pl}(\delta^M - 1)\Big) + \frac{Q}{A_0 D_{ex}}\frac{\exp\Big(Pe_{g,pl}(\delta^M - 1)\Big)}{(2\beta)^M}\right]. \tag{S32}$$

Using Eq. S27, $K_2$ can be evaluated as

$$K_2 = c_{g,0}\exp(Pe_{g,pl}) - \frac{K_1}{Q}\exp(Pe_{g,pl}), \tag{S33}$$

Substituting the magnitudes of $K_1$ (Eq. S32) and $K_2$ (Eq. S32) in Eq. S24 provides us with the following expression

$$\frac{c_{g,N}}{c_{g,0}} = \frac{\left[1 - \frac{c_{g,\infty}}{c_{g,0}}\exp\Big(Pe_{g,pl}(\delta^M - 1)\Big))\right]\left[1 - \exp\Big(-Pe_{g,pl}(\delta^N - 1)\Big)\right]}{\left[1 - \exp\Big(Pe_{g,pl}(\delta^M - 1)\Big) + Z\frac{\exp\Big(Pe_{g,pl}(\delta^M - 1)\Big)}{(2\beta)^M}\right]} + \exp\Big(-Pe_{g,pl}(\delta^N - 1)\Big), \tag{S34}$$

which can be re-arranged considering $c_{g,\infty} \to 0$ to give the final form of Eq. S24 as

$$\lim_{c_{g,\infty}\to 0} \frac{c_{g,N}}{c_{g,0}} = \phi_{g,N} = 1 + \frac{\left[\exp\Big(Pe_{g,pl}(\delta^N - 1)\Big) - \exp\Big(Pe_{g,pl}(\delta^M - \delta^N)\Big)\right]\left[1 - \frac{Z}{2\beta^M}\right]}{1 - \exp\Big(Pe_{g,pl}(\delta^M - 1)\Big) + Z\frac{\exp\left(Pe_{g,pl}(\delta^M - 1)\right)}{(2\beta)^M}}. \tag{S35}$$

## S3. SUPPORTING RESULTS

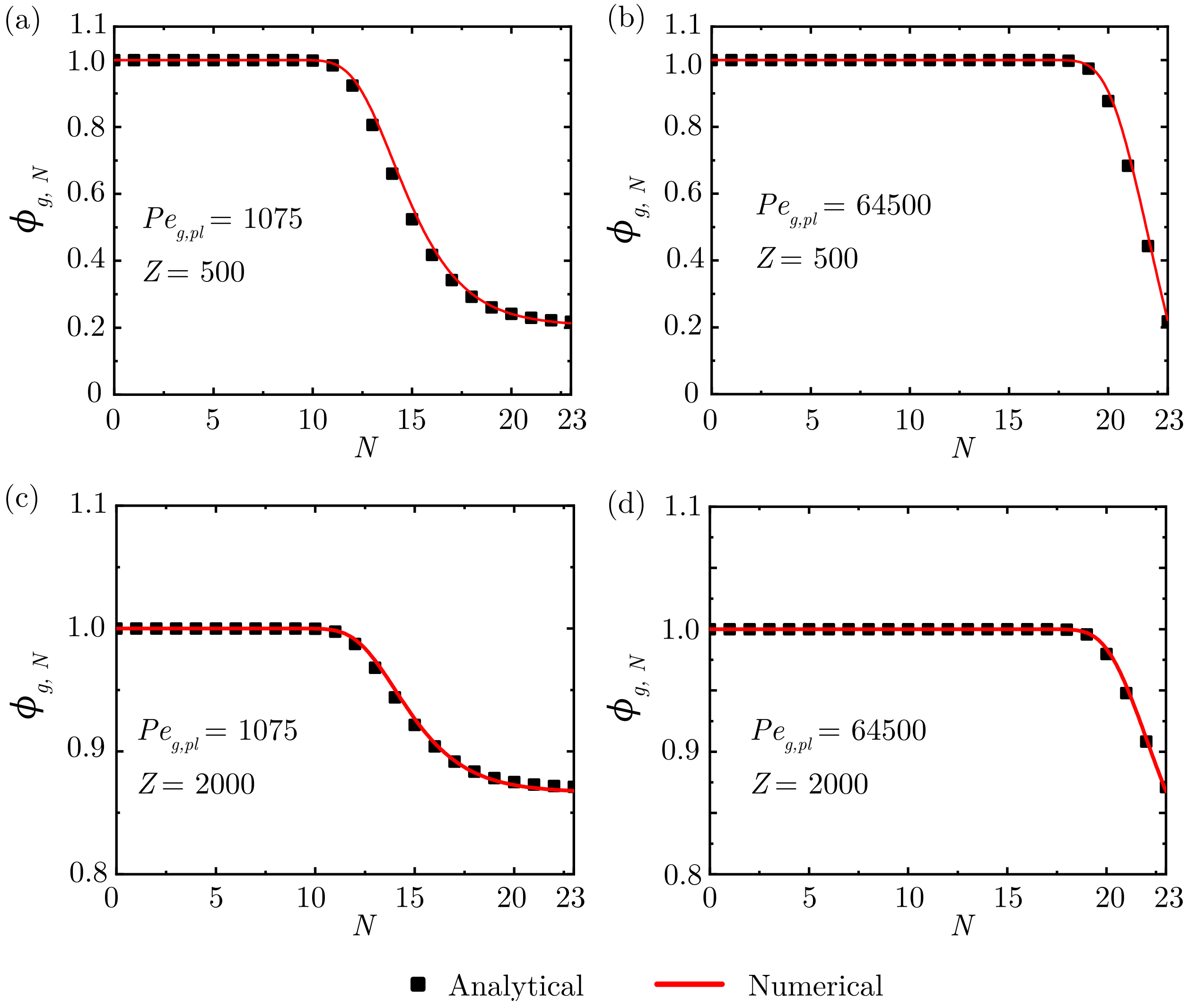


FIG. S1. Comparison of numerical prediction of steady-state $\phi_{g,N}$ with the analytical prediction (using the power-law model) for different combinations of $Pe_{g,pl}$ and $Z$ covering the entire range of the parameters considered in this analysis. The results are shown considering $St_a = 0.01$, $\alpha = 0.73, \beta = 0.71$. Note the change in scales between the top row and bottom row figures.

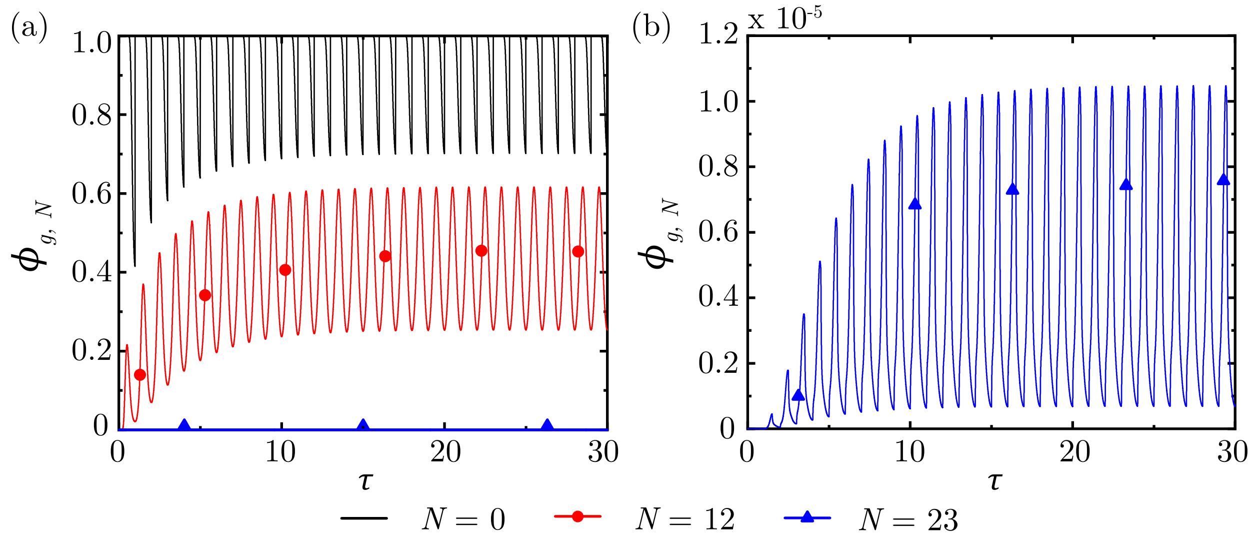


FIG. S2. Temporal change in $\phi_{g,N}$ at different lung generations for a physiologically realistic cyclic flow situation and considering a continuous gas source at the entrance to trachea. The results are shown for $Pe_g = 64500$, $Z = 500$, $St_a = 0.01$, $\alpha = 0.73, \beta = 0.71$

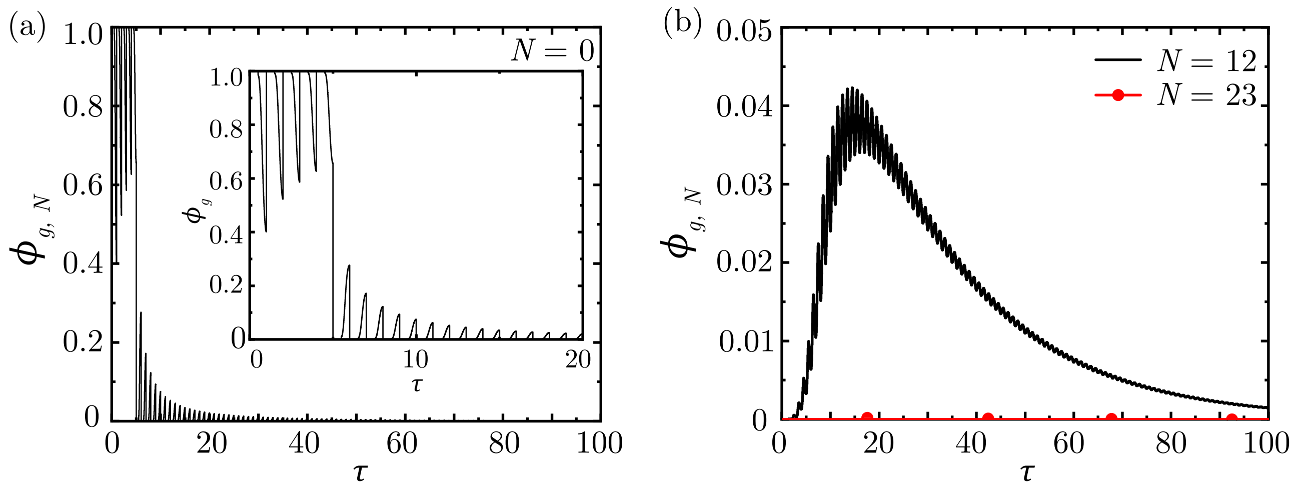


FIG. S3. Temporal change in $\phi_{g,N}$ at different lung generations for a physiologically realistic cyclic flow situation and considering finite availability of gas source at the entrance to trachea. The results are shown for $Pe_g = 64500$, $Z = 500$, $St_a = 0.01$, $\alpha = 0.73, \beta = 0.71$ and $\tau_{exp} = 5$.

## REFERENCES

[1]E. R. Weibel, A. F. Cournand, and D. W. Richards, *Morphometry of the human lung*, Vol. 1 (Springer, 1963).

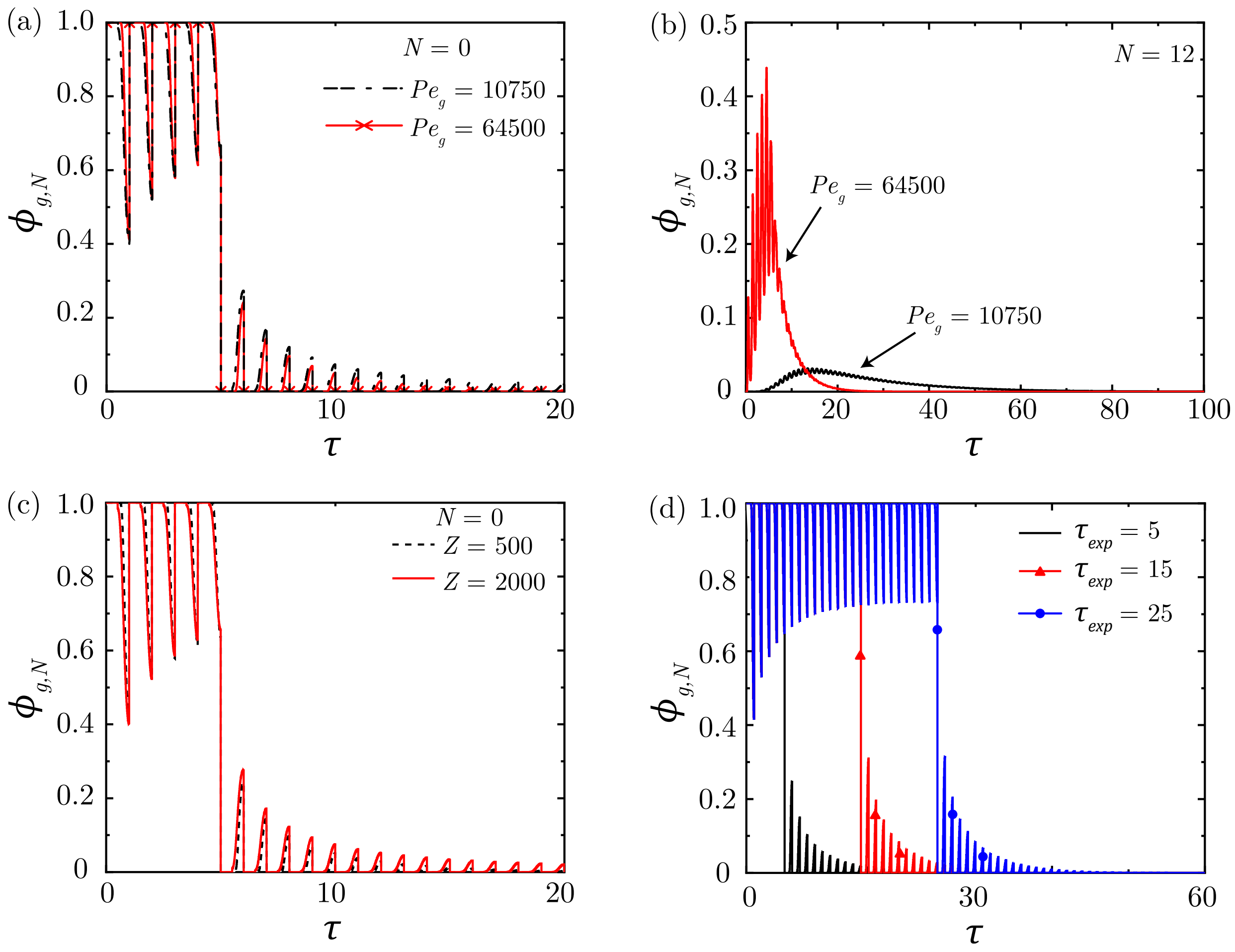


FIG. S4. Temporal change in $\phi_{g,N}$ for two different $Pe_g$ considering $Z = 500$, $St_a = 0.0095$ and $\tau_{exp} = 5$ at (a) $N = 0$ and (b) $N = 12$. (c) Temporal change in $\phi_{g,N}$ for two different $Z$ considering $Pe_g = 64500$, $St_a = 0.0095$ and $\tau_{exp} = 5$ at $N = 0$ (d) Temporal change in $\phi_{g,N}$ for three different $\tau_{exp}$ considering $Pe_g = 64500$, $St_a = 0.0095$ and $Z = 500$ at $N = 0$. The results are shown for $\alpha = 0.73, \beta = 0.71$.


[2]A. Chakravarty, M. V. Panchagnula, A. Mohan, and N. A. Patankar, "Pulmonary drug delivery and retention: A computational study to identify plausible parameters based on a coupled airway-mucus flow model," PLOS Computational Biology **18**, e1010143 (2022).

[3]A. Chakravarty, M. V. Panchagnula, and N. Patankar, "Inhalation of virus-loaded droplets as a clinically plausible pathway to deep lung infection," Frontiers in Physiology **14**, 18 (2023).

[4]D. Kundu and M. V. Panchagnula, "Asymmetric lung increases particle filtration by deposition," Scientific Reports **13**, 9040 (2023).

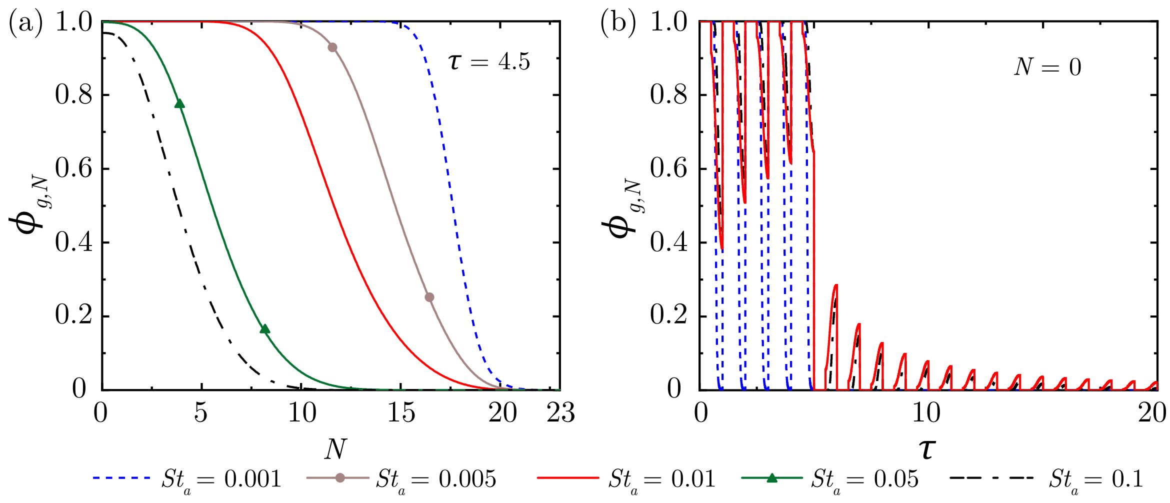


FIG. S5. (a) Progression of $\phi_{g,N}$ into the lung for various $St_a$ at $\tau = 4.5$ and (b) Temporal change in $\phi_{g,N}$ at $N = 0$ for various $St_a$. The results are shown for $Pe_g = 64500$, $Z = 2000$, $\tau_{exp} = 5$, $\alpha = 0.73, \beta = 0.71$.

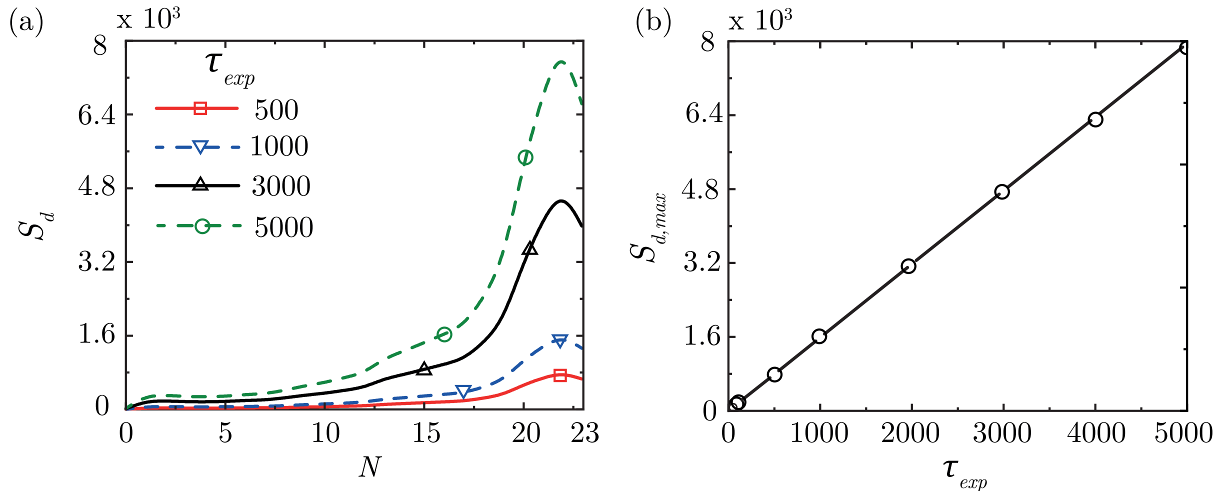


FIG. S6. (a) Total deposition ($S_d$) of particles within the lung at the end of exposure for different exposure duration ($\tau_{exp}$) and (b) Increase in maximum particle deposition ($S_{d,max}$) with rise in exposure time. The results are shown for $Pe_{p,a} = 2.85 \times 10^{10}$ and $St_a = 0.0095$. Figure reproduced with permission from Chakravarty et al.[2].

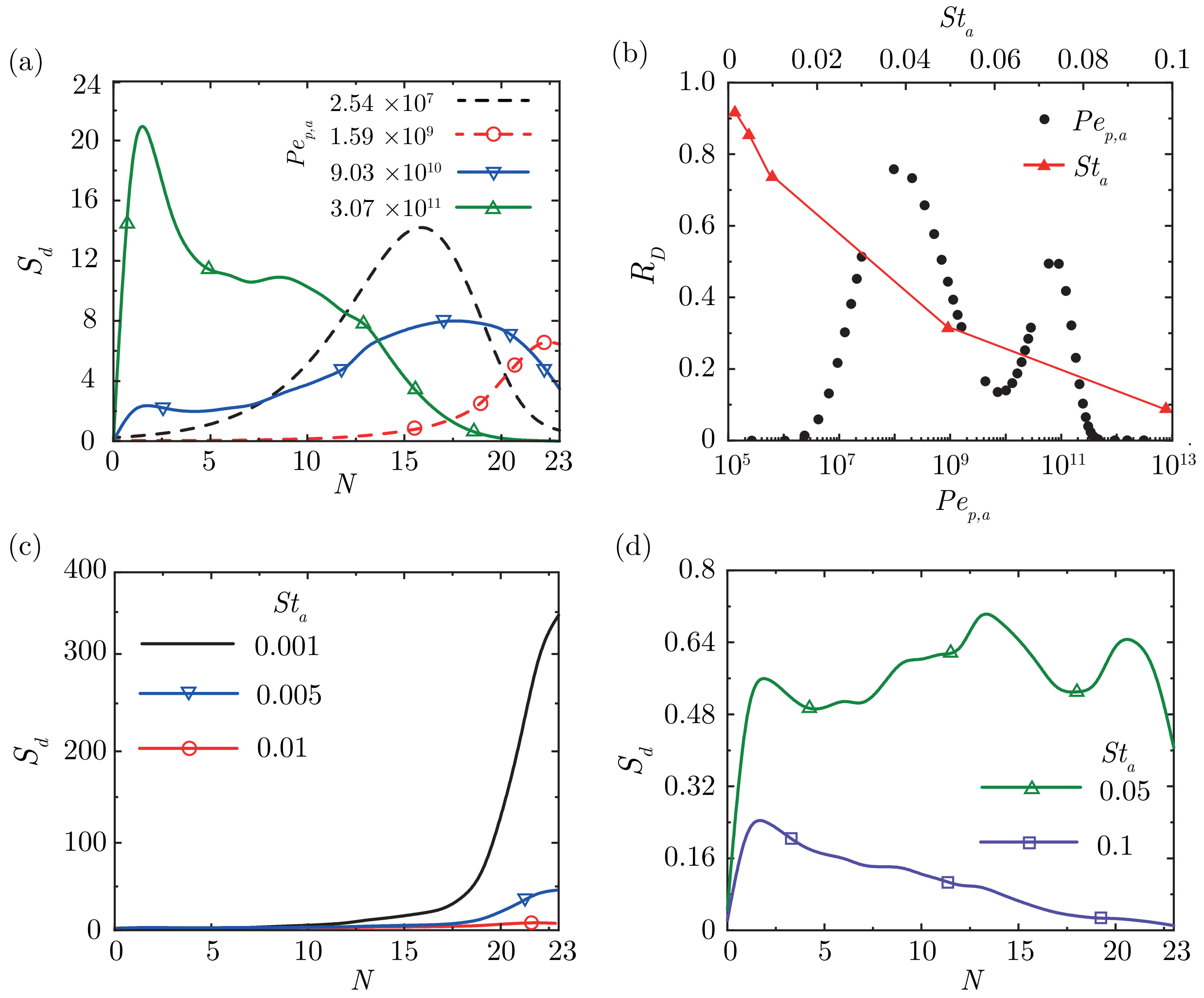


FIG. S7. (a) Total particle deposition ($S_d$) within the lung for different $Pe_{p,a}$ considering $St_a$ = 0.0095 and $\tau_{exp}$ = 5.(b) Ratio ($R_D$) of particles deposited in the deep lung ($N > 17$) to that deposited in the whole lung with change in $Pe_{p,a}$ and $St_a$ (c-d) Total particle deposition ($S_d$) within the lung for different $St_a$ considering $Pe_{p,a} = 2.85 \times 10^{10}$ and $\tau_{exp} = 5$. Figure reproduced with permission from Chakravarty et al.[2].

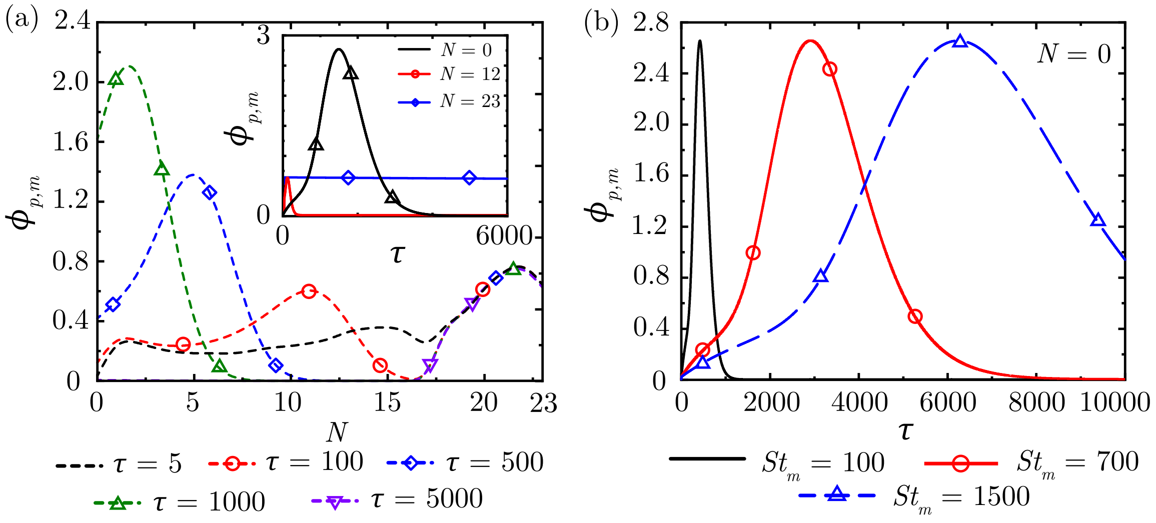


FIG. S8. (a) $\phi_{p,m}$ within the lung at different time instances and (inset) variation of $\phi_{p,m}$ with time at three different lung generations ($N = 0, 12, 23$) considering $Pe_{p,a} = 2.85 \times 10^{10}$, $St_a = 0.0095$, $Pe_{p,m} = 4.56 \times 10^7$, $St_m = 359.7122$, $\tau_{exp} = 5$. (b) Temporal change in $\phi_{p,m}$ at $N = 0$ for different $St_m$ other parameters remaining same. Figure reproduced with permission from Chakravarty et al.[2].

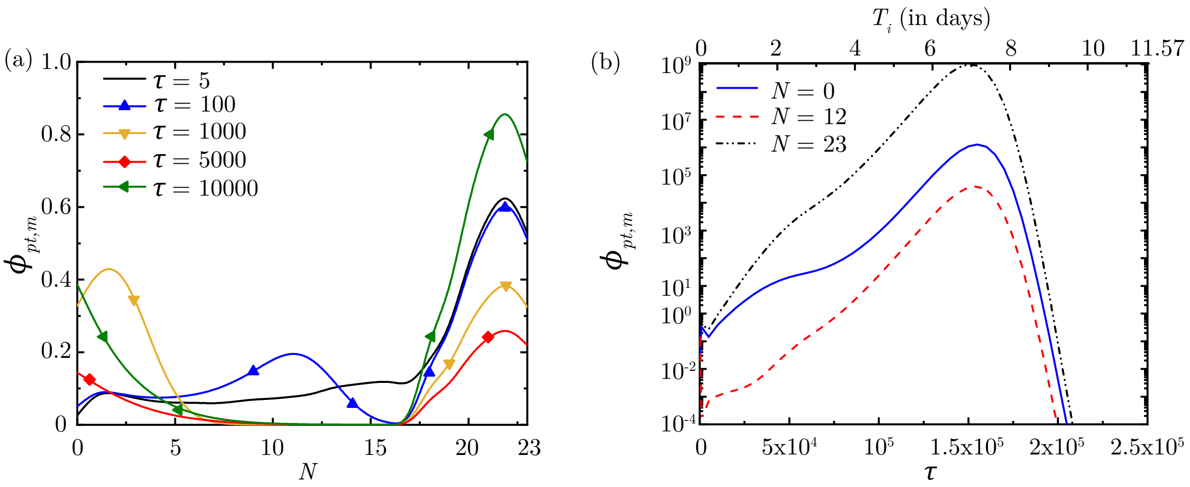


FIG. S9. (a) Dimensionless pathogen concentration in mucus ($\phi_{pt,m}$) within the LRT at different dimensionless time instances post infection onset and (b)Temporal change in $\phi_{pt,m}$ at different spatial locations within the LRT ($N$ is the lung generation). The results are shown for a SARS-CoV-2 infection. The results in (b) are also shown with respect to a dimensional time ($T_i$) post infection onset considering breathing time period ($T_b = 4$s). Figure reproduced with permission from Chakravarty et al.[3].

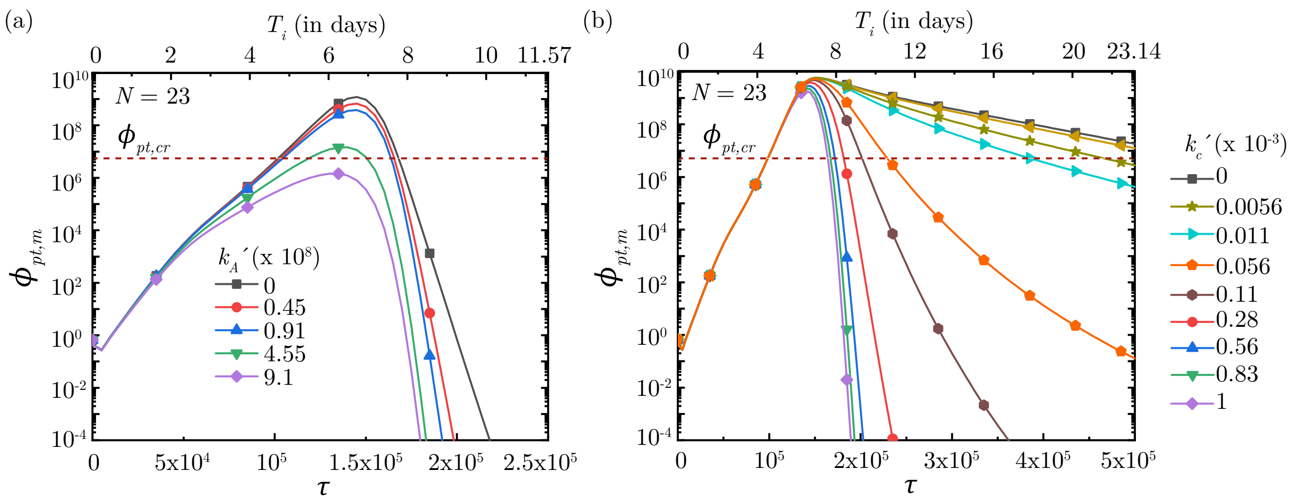


FIG. S10. Temporal change in pathogen concentration ($\phi_{pt,m}$) at $N = 23$ (deep lung) with change in (a) binding affinity of antibodies ($k'_A$) and (b) the rate at which cytotoxic T-lymphocytes eliminate the infected cells ($k'_c$). The results are shown for SARS-CoV-2 infection with respect to dimensionless time ($\tau$) as well as a dimensional time ($T_i$) post infection onset with the breathing time period ($T_b = 4$s). The dotted line indicates the critical SARS-CoV-2 load in the deep lung required for pneumonia onset. Figure reproduced with permission from Chakravarty et al.[3].

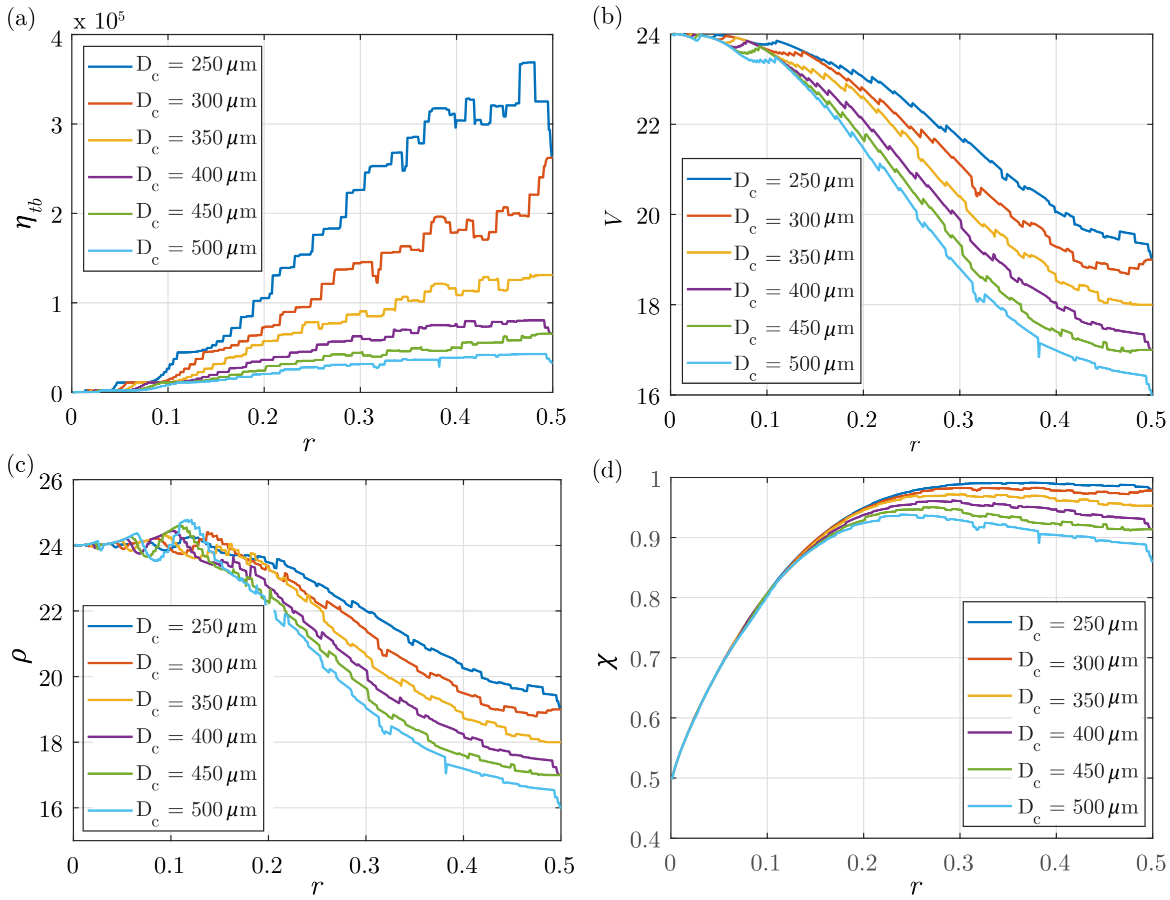


FIG. S11. Variation of (a) number of terminal bronchioles ($\eta_{tb}$), (b) volume occupied by the bronchial tree ($V$), (c) resistance offered to fluid flow ($\rho$) and (d) particle filtration efficiency ($\chi$) as function of degree of asymmetry ($r$) for different values of cut-off diameter ($D_c$). Reproduced with permission from Kundu et al.[4].
